# Effect of complementary feeding behavior change communication delivered through community-level actors on dietary adequacy of infants in rural communities of West Gojjam Zone, Northwest Ethiopia: A cluster-randomized controlled trial

**DOI:** 10.1371/journal.pone.0238355

**Published:** 2020-09-03

**Authors:** Chalachew Abiyu, Tefera Belachew

**Affiliations:** 1 School of Medicine, College of Medicine and Health Sciences, Wollo University, Dessie, Ethiopia; 2 Faculty of Public Health, Department of Nutrition and Dietetics, Jimma University, Jimma, Ethiopia; University of Ghana, GHANA

## Abstract

**Background:**

Attaining the recommended level of adequacy of the infant’s diet remains a serious challenge in most developing countries. Complementary foods, particularly in developing countries, are inadequate in quality and quantity that can result in adverse health and nutrition consequences in infants. This could be not only because of lack of food but also associated with caregiver’s poor knowledge, harmful cultural norms and behaviors on infant feeding. The promotion of optimal complementary feeding through behavior change interventions is a global health priority. However, many of the interventions targeted only mothers/caregivers of infants, and studies that engaged other family members are limited worldwide. Moreover, such interventions are scarce in developing countries, including Ethiopia. This trial aimed to evaluate the effectiveness of complementary feeding behavior change communication delivered through community-level actors on the dietary adequacy of infants.

**Methods:**

We conducted a cluster-randomized controlled trial in rural communities of West Gojjam Zone, Northwest Ethiopia. Trial participants in the intervention clusters received complementary feeding behavior change communication for 9 months whereas those in the control clusters received only the usual care. Trained women development army leaders delivered the intervention. A pre-tested, structured interviewer-administered questionnaire was used for data collection. Generalized estimating equations regression analyses adjusted for baseline covariates and clustering were used to test the intervention effects.

**Results:**

The intervention showed positive statistically significant effects on the consumption of dairy products [RR = 1.8; 95% CI: 1.04–3.13], eggs [RR = 3; 95% CI: 1.35–6.56], vitamin A-rich fruits and vegetables [RR = 2.7; 95% CI: 1.17–6.1], other fruits and vegetables [RR = 5; 95% CI: 2.49–10.58] and animal-source foods [RR = 2; 95% CI: 1.39–2.87]. The proportions of infants who achieved minimum dietary diversity [RR = 3; 95% CI: 1.34, 7.39], minimum meal frequency [RR = 2.4; 95% CI: 1.37–4.29], and minimum acceptable diet [RR = 2.7; 95% CI: 1.13–7.23] were significantly higher in the intervention as compared to control groups.

**Conclusions:**

Complementary feeding behavior change communication delivered through community-level actors significantly improved the dietary adequacy of infants.

**Trial registration:**

ClinicalTrials.gov, NCT03488680. Registered 5 April 2018- Retrospectively registered, https://clinicaltrials.gov/ct2/show/NCT03488680.

## Introduction

Most events of malnutrition happen in the first 2 years of life when there is a high demand for adequate diets [1]. Malnutrition during this period will cause irreversible damage in physical growth, brain development, and morbidity and mortality. A malnourished girl could give birth to a malnourished baby [2].

Malnutrition in children under five years of age remains a major public health problem in Ethiopia. The prevalence of stunting, underweight and wasting were 38%, 24%, and 10%, respectively in children under five years of age [3]. The study area particularly has a high rate of malnutrition even though the area is a surplus crop producer. This could be due to suboptimal child feeding practices which is in turn related to poor knowledge and unfavorable attitude towards feeding practices [4].

From all known health and nutrition preventive intervention strategies, optimal infant and young child feeding (IYCF) has the best significant impact on child growth and survival [5]. Globally, suboptimal feeding practices account for more than half of the deaths of under five children. Over two-thirds of these deaths are related to inappropriate feeding practices during the first 2 years of life [6].

Previous studies revealed that younger maternal age, unemployment, absence of formal education, low antenatal care visits, food insecurity and being in rural areas were identified as factors associated with suboptimal child feeding practices [7]. Moreover, lower postnatal care visits, young infant age, lower birth order, poor household wealth status, place of delivery and insufficient maternal exposure to mass media (television, radio, or newspapers) were the factors associated with inappropriate feeding practices in developing countries [8].

According to WHO, optimal complementary feeding refers to the introduction of safe, age-appropriate, and nutritionally adequate foods in addition to breastfeeding for infants at 6 months of age [9]. Suboptimal complementary feeding practices have been widely reported in Ethiopia [10]. The optimal complementary feeding practice is only 7%. About 56% of infants aged 6–8 months consumed complementary foods, 45% of infants were fed the minimum meal frequency and 14% of children achieved the minimum dietary diversity [3].

The complementary feeding period is a critical time of transition in infants characterized by a gradual shift from breast milk to family food. The incidence of growth faltering increases significantly at 6 months of age when complementary foods are being introduced particularly in most low and middle-income countries including Ethiopia [11]. Suboptimal complementary feeding practices are associated with a high burden of malnutrition and mortality in infants [12].

Diets adequate in diversity and quantity are important for the provision of essential nutrients to infants [13]. Attaining the recommended level of diversity and adequacy of an infant’s diet remains a serious challenge in many developing countries including Ethiopia. The quality of an infant’s diet is dependent on food items contained in the diet and meal frequency [14]. Complementary foods are mainly starchy-staples that lack the desired quality, amount, and nutrient density for optimal child growth and development of infants. They are also initiated untimely (too early or too late) and prepared in an unhygienic way [4].

Inappropriate complementary feeding practices, with their negative health consequences, remain a significant public health problem worldwide [15]. Promoting optimal complementary feeding practices is a global health priority to improve infant feeding practices particularly in developing countries [16]. The Ethiopian government carried out several efforts to enhance complementary feeding practices at different times through the implementation of IYCF guideline across the country. However, these efforts failed to improve feeding practices at the expected level [17].

Suboptimal complementary feeding practices are not only caused by the lack of food, but also associated with poor knowledge, attitude, harmful cultural norms and behaviors of mothers/caregivers. Behavior change interventions are necessary to provide the appropriate information for mothers and change their feeding behaviors [18].

Community-based behavior change interventions have been employed in different parts of the world on an individual or group basis, through health facilities or home visiting programs, using printed materials such as leaflets, counseling, teaching sessions, peer support, videos, and practical demonstrations. Educational interventions were delivered for caregivers’ by health and nutrition officers in Indonesia [19], by primary health care providers in rural China [20], through the health services in Peru [21]. However, many of the interventions targeted only mothers/caregivers and studies that engaged other family members are limited worldwide. Moreover, such interventions are scarce in developing countries including Ethiopia.

In Ethiopia, a few behavior change interventions aimed at improving the IYCF practices have been conducted by Non-governmental organizations (NGOs) projects [22–25]. The reports of these projects focus either on implementation fidelity [22] or are implementation research [23], and broad in scope, focusing not only on complementary feeding but also on other IYCF practices [24–26]. Moreover, none of the interventions targeted on age-specific complementary feeding practices, engaged community-level actors and delivered before infants entered the complementary feeding period (before 6 months). None of the projects also included control groups except a trial conducted in Hula woreda, Southern Ethiopia [25].

This trial aimed to evaluate the effectiveness of complementary feeding behavior change communication delivered through community-level actors in improving the dietary adequacy of infants. We hypothesized that complementary feeding behavior change communication delivered through community-level actors is superior to the routine health and nutrition care in improving the dietary adequacy of infants. This study was part of a larger study entitled “effectiveness of complementary feeding behavior change communication delivered through community-level actors in improving feeding practices, health, and growth of infants in West Gojjam Zone, Northwest Ethiopia” registered at clinicaltrials.gov as NCT03488680.

## Methods

### Study setting

This trial was conducted in rural communities of West Gojjam Zone, Ethiopia from February 2017 to March 2018. West Gojjam Zone is one of the 13 administrative zones of the Amhara regional state. It has 13 rural districts, and each district is divided into *kebeles*, the lowest administrative units in Ethiopia. According to the population projection of Ethiopia for all regions at the district level from 2014 to 2017, which is based on the 2007 national census, the zone has a total population of 2,560,131in 2016; of whom 1,262,144 were male and 1,297,987 were female. The rural part accounts for 92% of the total population. A total of 480,255 households were counted in this Zone, which results in an average of 4.39 persons to a household, and 466,491 housing units. From the total population mentioned, 315,228 were children of under five years of age of whom 160, 214 were under two years of age [27].

In the Amhara region, a total of 117,428 Health Development Army (HDA) groups and 532,259 one-to-five networks were established in 2011 [28]. The one-to-five networks are women volunteers who are empowered as an HDA to transform their society. They are trained to focus more intensively on sparking local behavior change making regular rounds to check on neighbors and encourage healthy lifestyles. They are from “model families” and serve as living examples that the health extension workers (HEW) messages are being heard [29]. The proportion of women of childbearing age is 24% [30].

### The context

The Ethiopian government started the Health Development Army (HDA) in 2011 intending to consolidate the gains made by the health extension programs (HEP) and promote community ownership of the programs. Although some regions have both male and female HDAs, HDAs are now basically women known as the women development army (WDA) [30].

WDA leaders are selected from the model families. A household that implemented all of the government's 16 priority health interventions, from vaccinating their children and sleeping under mosquito bed-nets to building separate latrines and using family planning, is recognized as a model family [31]. “Model families” are selected by HEW in collaboration with the *kebele* administration. They get certificates, are celebrated at *kebele* ceremonies and asked to support five other households in adopting the priority interventions [32]. WDAs leaders are unpaid health volunteers that undertake various preventive and promotive health services supported and supervised by HEWs [33].

Once the WDA groups are formed through participatory community involvement, the WDA leaders provided an intensive 7 to 10 days training [30], whose primary objective is to educate and mobilize the communities to utilize the maternal, neonatal and child health services delivered by the health post and health centers [34]. On average, there are approximately 30 WDA team leaders and 200 WDA network leaders in each *kebele* [30].

Each WDA group comprised 25–30 households (women) which are further organized into the “1 to 5” network of women where a model woman leads five other women within her neighborhood [35]. The one-to-five network functions as a forum for the exchange of concerns, priorities, problems, and decisions related to the health status of women. While being supported by the health extension workers (HEW), the networks are responsible for the preparation of plans and ensuring their completion, for the collection of health information, and also for conducting a weekly meeting to review progress and submitting monthly reports [36]. The WDA groups thus support the implementation of the HEP.

The one-to-five networks meet every week, while the larger health development team meets once every two weeks. Moreover, they review their performance against their plan and evaluate each other on a monthly basis and give grades based on their performances. A performance report including the results (grades) is organized at the health development team level and sent to the HEW [30].

**Fig 1:**
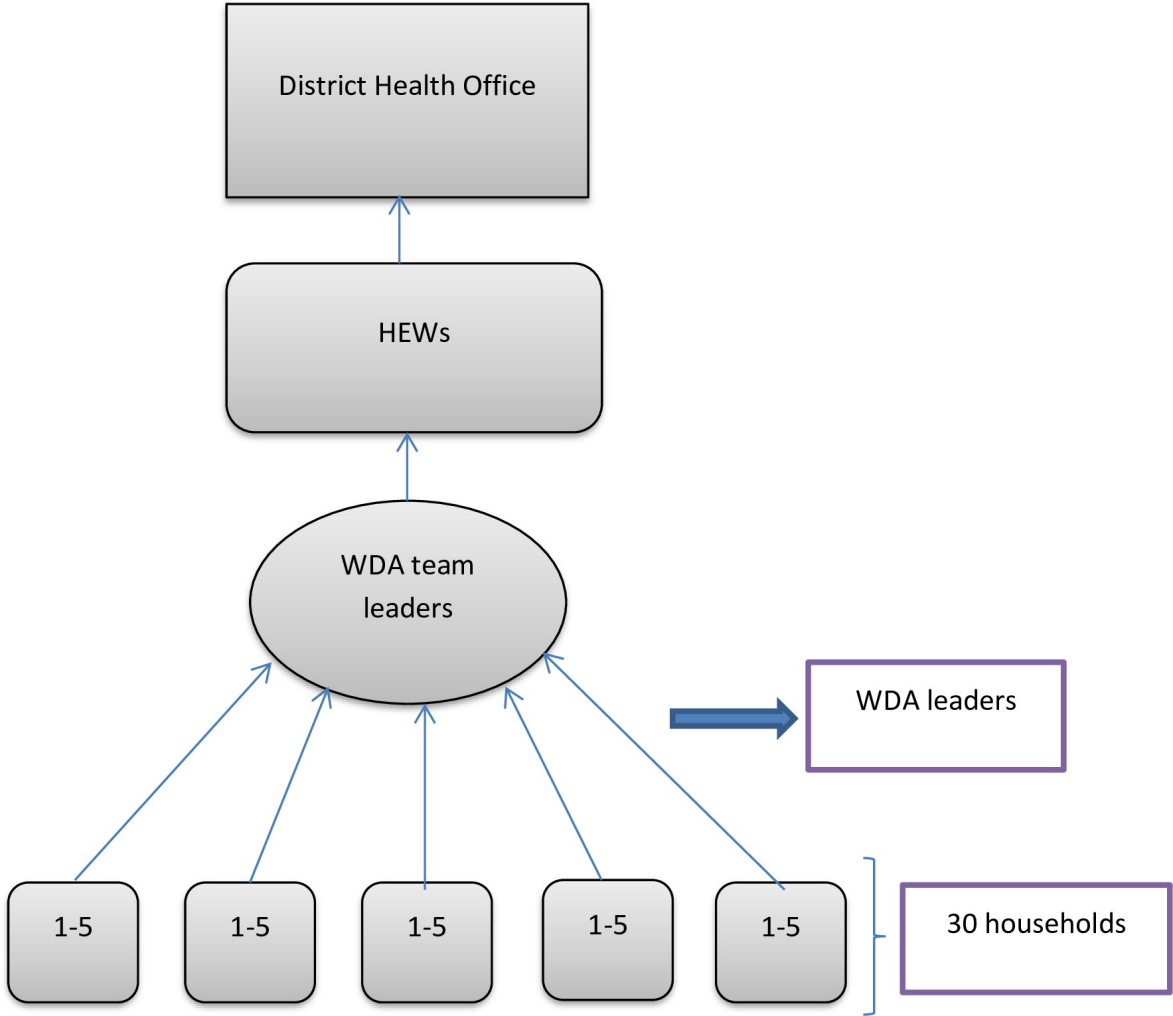
Hierarchy of WDA and reporting.

In our study context, community-level actors are those people living in the community who could influence change in harmful traditional feeding behaviors and provide a supportive environment for the adoption of the recommended feeding practices. These include WDA leaders and family members of the trial participants’ (Fig 1).

### Study design and population

**Fig 2:**
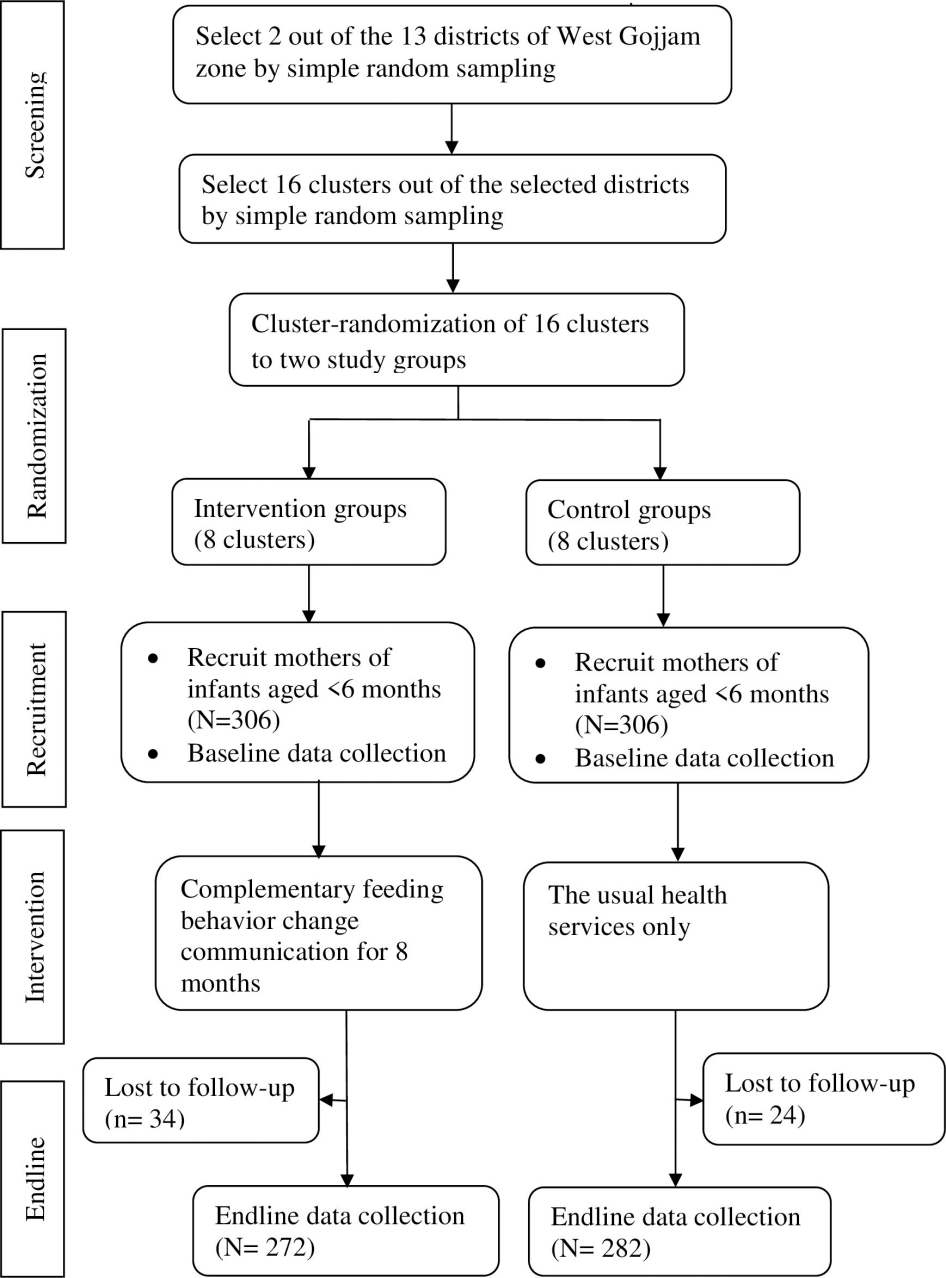
Trial profile.

A cluster-randomized controlled trial single-blind parallel-group, a two-arm trial with a 1:1 allocation ratio was carried out among mothers of infants aged <6 months of age at the time of enrolment. The trial was conducted in line with the CONSORT recommendations for cluster-randomized trials [37]. The intervention was delivered in community settings that encourage collective participation. Hence, the unit of randomization was clusters (*kebeles)* to minimize intervention contamination and facilitate logistical convenience. Consented mothers who were residents in the study area for at least 6 months before the commencement of the study were recruited and those who were ill and unable to communicate during the study were excluded (Fig 2).

### Sample size determination

One of the objectives of the larger study was to evaluate the effect of the intervention on complementary feeding practice (dietary adequacy) of infants. The sample size was calculated using *G-power* based on the following assumptions. Tail (s): One; Effect size d: 0.3; α error probability = 0.05; and power (1- β error probability) = 0.8. This gave a sample size of 278. Then, it was multiplied by the design effect of 2 [38], and allowing for a 10% loss to follow up, the total sample size was 612. The effect size of behavior change intervention on child linear growth was considered; the variable which gave the maximum sample size [39].

### Sampling and randomization

A two-stage cluster sampling technique was applied. First, 2 out of the 13 districts in West Gojjam zone were selected by simple random sampling (lottery method). Second, lists of all *kebeles* (clusters) in the selected districts were compiled from the district administrative offices. The number of study subjects in the selected clusters was obtained from the records of births prepared by HEW. Each cluster in the selected districts forms the sampling frame; while the mother-infant pairs within the cluster formed the final sampling units of observation. Simple randomization with a 1:1 allocation was used to assign clusters to either control or intervention groups. First, 16 non-adjacent clusters (that did not share geographic areas) were selected by simple random sampling. Then, the 16 clusters were listed alphabetically. A list of random numbers was generated in MS Excel 2010 and the generated values were fixed by copying them as “values” next to the alphabetic list of the clusters. These were arranged in ascending order according to the generated random number. Finally, the first 8 clusters were selected as intervention clusters and the last 8 as control clusters. A statistician that was blinded to study groups and not participated in the trial did the generation of the allocation sequence and the randomization of clusters.

### The intervention

Complementary feeding behavior change communication was delivered for only intervention clusters for 9 months whereas the control clusters received only the routine health and nutrition services. The language of communication during the intervention period was Amharic (the local language). The intervention had three parts.

#### Part 1: Training of women development army (WDA) leaders

A total of 24 WDA leaders who are influential members of their community were recruited by HEWs in the intervention clusters (3 in each cluster) and centrally trained on complementary feeding practices by the researcher qualified as nutritionist. The purpose of the training was to empower WDA leaders, who act as community-based IYCF counselors and support groups, with action-oriented knowledge, attitude, and behavior to effectively counsel, support and negotiate with mothers of infants and families to adopt recommended IYCF and health care practices.

The training sessions focused on the key messages on optimal complementary feeding practices followed by cooking demonstrations on how to prepare and integrate a variety of locally available foods into home-made complementary foods. The first training session was conducted at the beginning of the intervention whereas the second session with similar content was repeated at the middle (4 months) of the intervention period and each session lasted for 3 days. After training sessions, every participant received a copy of the visual materials (posters) containing the key messages for complementary feeding practices to be used as a reference. The training contents were adopted from the Alive and Thrive program in Ethiopia which are both language and culturally appropriate. Key messages were compiled into visual material (posters) used to deliver training [40].

Direct, interactive and participatory learner and activity-oriented instructional strategies were delivered. Talks, group discussions, group work exercises, demonstrations, role plays, storytelling, simulation, case studies and problem-solving were used to enhance knowledge, attitude, and behaviors. The intervention key messages were focused on the right time to introduce complementary foods; specific foods to be offered or avoided and how to offer them; meal frequencies; amounts of foods to be fed to infants at different ages while continuing breastfeeding; offering a variety of foods from different food groups; practice responsive feeding; practice good hygiene, and continue to feed the child during and after an illness (Table 1).

**Table 1:** Complementary feeding practices key messages in the intervention clusters.

No.	Key messages
1	Start feeding your baby soft and thick porridge made from a combination of cereal flours at 6 months. Continue breastfeeding up to 2 years and beyond.
2	Enrich baby’s porridge by adding one or more ingredients from animal-source foods (milk, egg, dried meat powder), finely chopped vegetables (kale, carrot, cabbage, tomato, potato) and mashed fruits (avocado, papaya, mango, banana, pumpkin) in each meal.
3	Cook and feed animal-source foods (e.g. eggs, beef, pork, chicken, liver, fish) at least 3 times per week. Feed your child fruits (e.g. ripe banana, mango, orange, papaya, avocado) after a meal at least once per day.
4	Increase variety, amount and frequency of feeding with age for the baby. Amount of food per meal: Begin with 2 to 3 tablespoons at 6 months of age. 2 to 3 tablespoonfuls and increase gradually to half (½) 250 ml cup at 6–9 months. Half (½) of 250 ml cup at 9–12 months. Three-quarters (¾) to 1 of 250 ml cup at 12–24 months. Frequency of feeding per day: 2–3 times at 6–8 months, 3–4 times at 9–23 months. Feed 1–2 snacks (e.g. sliced bread, fruits) between two major meals.
5	Encourage your baby to eat with patience and love. Don’t force your baby to eat. Provide extra food during and after an illness.
6	Feed your baby using a clean cup and spoon; and avoid bottle feeding. Wash your hands with soap and water before preparing food, and before feeding young children.
7	Enriched baby’s porridge preparation: • Prepare a germinated flour made up of 3/4^th^ staples (one or more ingredients from maize, wheat, rice, millet, sorghum, oat) and 1/4^th^ legumes (one or more ingredients from beans, lentils, chickpeas, groundnuts). • Use milk instead of water for preparing porridge. • Add butter/oil which will make the thick porridge easier to eat. • Add finely chopped meat, fish or eggs. • Add one or more ingredients from finely chopped vegetables and mashed fruits. • Increase the consistency and thickness of the porridge with child age. • Do not forget to use iodized salt.

#### Part 2: Group training of mothers by WDA leaders

Each member of the trained WDA leaders was assigned to 10–15 mothers with children aged younger than 6 months residing in their cluster/village. WDA leaders delivered a total of nine group training sessions including cooking demonstrations (once per month, for 3 days duration each) for the mothers they are assigned with the same training procedures provided by the researcher in part 1. WDA leaders applied culturally appropriate training sessions with mothers using posters prepared in the local language.

#### Part 3: Home visits

Each WDA leaders conducted a total of nine home visits (once per month, for 2 days duration each) in the intervention clusters that aimed to bring behavior change at maternal and family level. During each home visit, individual counseling and support were offered for each mother to reinforce the adoption of feeding practices she had been taught during the group training sessions, to observe feeding practices, to demonstrate cooking procedures, to correct the harmful practices and to provide appropriated feedback focusing on the key complementary feeding recommendations. A participatory discussion was held with family members (fathers and grandmothers of the recruited infants) regarding optimal complementary feeding practice, its impact on children’s nutrition and health; and how can they support the mother in feeding the baby. Each mother provided a poster containing the key messages at the end of each home visit.

All the activities of WDA leaders were supervised by HEWs and the overall supervision was done by the researcher. All activities done during the study are presented in (Table 2). Recruitment of study participants and baseline data collection was conducted between February and March 2017. Following the baseline survey, the intervention was delivered for the intervention clusters from April 2017 to December 2017. The endline data collection was carried out between January and February 2018.

**Table 2:** Schedule of activities during the study period.

Activities	Time points in months												
	1	2	3	4	5	6	7	8	9	10	11	12	13
Enrollment and baseline data collection	x^**I+C**^	x^**I+C**^											
Training of WDA leaders			x^**I**^				x^**I**^						
Group training of mothers			x^**I**^	x^**I**^	x^**I**^	x^**I**^	x^**I**^	x^**I**^	x^**I**^	x^**I**^	x^**I**^		
Home visits			x^**I**^	x^**I**^	x^**I**^	x^**I**^	x^**I**^	x^**I**^	x^**I**^	x^**I**^	x^**I**^		
Process evaluation			x^**I**^	x^**I**^	x^**I**^	x^**I**^	x^**I**^	x^**I**^	x^**I**^	x^**I**^	x^**I**^		
Endline data collection												x^**I+C**^	x^**I+C**^
Supervision	x^**I+C**^	x^**I+C**^	x^**I**^	x^**I**^	x^**I**^	x^**I**^	x^**I**^	x^**I**^	x^**I**^	x^**I**^	x^**I**^	x^**I+C**^	x^**I+C**^

^**I**^Intervention groups; ^**C**^Control groups; ^**I+C**^Activities both in intervention and control groups.

#### Blinding

Data collectors were not informed of the allocation clusters and were not residents in any of the clusters. However, trial participants knew the intervention allocation due to the nature of the intervention.

#### Process evaluation

Process evaluation was conducted to document the intervention implementation process and assess whether the intervention activities were implemented as planned, evaluate the performance of WDA leaders and the extent to which the intervention reached the intended mothers and family members (Table 3).

**Table 3:** Process evaluation.

Data sources	Process indicators	Characteristics
**1. Assess whether the intervention activities are implemented as planned**		
Activity logs	• Number of training sessions including cooking demonstrations held with WDA leaders • Number of visual materials distributed to WDA leaders • Number of training sessions including cooking demonstration held with mothers • Number of visual materials distributed to mothers	Fidelity
**2. Evaluate the performance of WDA leaders**		
Attendance records	• Number of recruited WDA leaders • Number of WDA leaders trained • Number of home visits conducted by WDA leaders	Dose delivered (exposure)
**3. Evaluate the extent to which the intervention reached the intended mothers and family members**		
Attendance records	• Number of recruited mother-infant pairs • Number of mothers trained • Number of mothers attended home visits • Number of family members attended home visits	Dose delivered (exposure)

### Data collection methods and outcome measurements

A baseline survey was conducted following enrolment of mothers using a pre-tested structured interviewer-administered questionnaire to assess the infant, maternal and household characteristics in both study groups at the same time. Mothers’ knowledge and attitude about complementary feeding were among the baseline data examined. Mothers’ knowledge on complementary feeding was computed based on six knowledge questions with “yes” or “no” responses. Likewise, mothers’ attitude towards complementary feeding was computed based on six attitude questions with “agree”, “disagree” and “don’t know” responses. A score of "1" was given for each correct response and "0" for the wrong response. The scores summed and mean scores for knowledge and attitude questions were computed. Listening “seven solutions”, a radio drama focused on infant and young child feeding, could affect mothers’ knowledge and attitude about complementary feeding, and their feeding practices. That is why the mothers were asked whether they listen “seven solutions” or not, and included in Table 4.

**Table 4:** Baseline characteristics of the study participants by study groups in rural communities of West Gojjam Zone, Northwest Ethiopia, 2017–2018.

Variable	Control group (N = 282)	Intervention group (N = 272)
**Child**		
Sex (%)		
Male	55.3	54.6
Female	44.7	45.6
Age (months), mean±SD	3.22±1.4	3.21±1.48
**Maternal**		
Age (months), mean±SD	27.2±5	28.05±4.8
Educational status (%)		
Attended formal education	23.8	19.4
No formal education	76.2	80.6
Occupation (%)		
Farmer	12.1	10.8
Housewife	87.9	89.2
Marital status (%)		
Single	2.1	1.8
Married	93.6	94.6
Divorced	3.1	2.6
Widowed	1.1	1
Parity (%)		
Primiparous	16.7	19.8
Multiparous	83.3	80.2
Perception of child's weight (%)		
Large	26.6	28.9
Medium	48.6	45.6
Small	24.5	25.3
ANC visit (%)		
Yes	73.4	71.4
No	26.6	28.6
Place of delivery (%)		
Home	63.5	64.8
Health facility	36.5	35.2
PNC checkup (%)		
Yes	27	22.7
No	73	77.3
IYCF counseling (%)		
Yes	33	30.4
No	67	69.6
Knowledge score on CFP, mean±SD	3.83±1.25	3.78±1.27
Attitude score on CFP, mean±SD	3.59±1.33	3.7±1.26
**Household**		
Family size, mean ± SD	5.5±1.8	5.3±1.9
Possession of radio (%)		
Yes	19.5	22
No	80.5	78
Listens to *seven solutions* (%)		
Yes	27.3	21.7
No	72.7	78.3

*CFP: complementary feeding practices.

*Seven solutions: a radio drama focused on infant and young child feeding (IYCF) practices.

At the end of the intervention period, endline data collection was carried out to examine complementary feeding practices of mothers for their infants in both the intervention and control groups at the same time. Recruited infants aged <6 months at baseline survey achieved 9–15 months of age at the time of endline data collection.

The key dietary adequacy indicators; minimum dietary diversity (MDD), minimum meal frequency (MMF) and minimum acceptable diet (MAD), were determined based on WHO guidelines [41]. The dietary intake of infants was determined based on the 24 hours dietary recall of mothers. The seven food groups used for determination of these indicators were: (i) grains, roots & tubers; (ii) legumes and nuts; (iii) dairy products; (iv) flesh foods (meat, poultry, and fish) (v) eggs; (vi) vitamin A-rich fruits and vegetables; and (vii) other fruits & vegetables. Each of the 7 food groups was allocated a score of 1 [41]. Data collectors applied different probing techniques to help mothers recall the number of meals and type of foods they provided for infants that would minimize the recall bias.

### Definition of terms

MDD: the proportion of infants 6–23 months of age who receive foods from four or more food groups during the previous day [41].

MMF: the proportion of infants 6–23 months of age who receive solid, semi-solid or soft foods the minimum number of times or more (minimum is defined as two times for breastfed infants 6–8 months; three times for breastfed children 9–23 months) in the previous day [41].

Minimum acceptable diet (MAD): the proportion of infants 6–23 months of age who had at least the minimum dietary diversity and the minimum meal frequency during the previous day [41].

### Data quality control

The questionnaire was prepared in English, and translated to the local language Amharic and then back to English by experts of the language to keep its consistency. Training on data collection tools was given to data collectors and supervisors. A pre-test was done on 5% of the sample to assess the clarity of the questions and applicability of the instrument. After the pre-test, some questions were reformed and re-ordered. To enhance blinding, precise objectives of the study and cluster allocation to the trial were not disclosed to data collectors and the data collection schedule was randomized. WDA leaders were not involved in the data collection. Daily supervision was conducted by the supervisors and the overall supervision was done by the researcher.

### Data processing and analyses

Data were double entered into the EPI-Info, exported to SPSS version 21 for cleaning and statistical analysis. Baseline differences between the study groups were tested using the chi-square test for categorical variables and *t*-test for continuous variables. Binary generalized estimating equations (GEE) regression analysis adjusted for potential covariates and clustering were used to test differences in dietary adequacy indicators between the intervention and control groups. All analyses were conducted according to the intention to treat (ITT) principle and the adjusted effect measures were considered as the main results. P-value <0.05 was considered as statistically significant.

### Ethical standards disclosure

All procedures involving the research were approved by Jimma University College of Health sciences institutional and review board in February 2016. Permission to undertake the study was obtained from the regional, zonal and district administration and health offices of the study area. After the identification of eligible mothers, the nature and purpose of the study were explained along with their right to refuse. Written informed consent was obtained from all study participants. The right of the participant to withdraw from the study at any time was respected. The data were not accessed by a third person, except the investigators, and were kept confidential. The trial was registered at clinicaltrials.gov as NCT03488680. The trial was registered after the enrolment of the study participants because the registration process taken longer time than we expected. The enrolment of the participants began before the trial was registered to conduct the trial according to the time plan. The authors confirm that all ongoing and related trials for this intervention are registered.

## Results

At baseline, a total of 612 mother-infant pairs (306 in the control and 306 in the intervention group) were recruited yielding a response rate of 100%. Of these, 34 (11%) in the intervention group and 24 (7.8%) in the control group were excluded in the study because they moved away from the study area, decided not to continue in the study or were lost to follow-up during the endline data collection. Overall, endline data were completed for 554 (90.5%) of the study participants in both study groups.

### Baseline characteristics

Baseline infant, maternal, and household characteristics were comparable between the intervention and control clusters (Table 4).

### Effects of the intervention

The consumption frequencies of food items used for measuring infants and young children’s dietary adequacy indicators recommended by WHO are presented in Table 5. The intervention showed positive effects on the food items consumed ranging from 2 to 27 percentage points with statistically significant differences in the consumption of dairy products, eggs, vitamin A-rich fruits and vegetables, other fruits and vegetables and all animal-source foods.

**Table 5:** Generalized estimated equation regression analyses on the type of food groups consumed by infants at endline by study groups in rural communities of West Gojjam Zone, Northwest Ethiopia, 2017–2018.

Food items	Study groups	n (%)	*RR (95% CI)
Grains, roots & tubers	CG	221 (88.4)	1
	IG	235 (90.7)	1.1 (0.451–2.647)
Legumes & nuts	CG	60 (24.0)	1
	IG	138 (28.6)	1.1 (0.451–2.647)
Dairy products	CG	94 (37.6)	1
	IG	138 (53.3)	1.8 (1.044–3.128)
Flesh foods	CG	11 (4.4)	1
	IG	17 (6.6)	1.2 (0.326–4.636)
Eggs	CG	24 (9.6)	1
	IG	66 (25.5)	3 (1.347–6.558)
Vitamin A-rich fruits & vegetables	CG	23 (9.2)	1
	IG	59 (23.0)	2.7 (1.173–6.1)
Other fruits & vegetables	CG	36 (14.4)	1
	IG	84 (32.4)	5 (2.491–10.579)
Animal-source foods	CG	96 (38.4)	1
	IG	142 (54.8)	2 (1.392–2.869)

CG: control group (N = 250); IG: intervention group (N = 259); RR: relative risk; CI: confidence interval.

*RRs were as yielded by the “Generalized estimated equation regression analyses.”

The proportions of infants who consumed grain, roots, and tuber were almost similar between the control group, 88.4%, and intervention groups, 90.7%, [RR = 1.1; 95% CI: 0.45–2.65]. Likewise, there was no significant difference in the percentage of legumes and nuts consumed; 24% in the control and 28.6% in the intervention groups, [RR = 1.1; 95% CI: 0.45–2.65]. There was also no statistically significant difference in the consumption frequencies of flesh foods between the study groups; 4.4% in the control and 6.6% in intervention groups, [RR = 1.2; 95% CI: 0.33–4.64].

On the other hand, the intervention showed a positive statistically significant effect on the proportion of dairy products consumed; 53.3% in the intervention and 37.6% in the control group, [RR = 1.8; 95% CI: 1.04–3.13]. The consumption of eggs was higher in the intervention group, 25.5% as compared to the control group, 9.6%, and the difference was statistically significant, [RR = 3; 95% CI: 1.35–6.56]. Similarly, the intervention significantly influenced consumption frequency of vitamin A-rich fruits and vegetables; 9.2% in the control and 23% in intervention group, [RR = 2.7; 95% CI: 1.17–6.1]. Likewise, there was a statistically significant improvement in the consumption frequency of other fruit and vegetables; 14.4% in the control and 32.4% in the intervention group, [RR = 5; 95% CI: 2.49–10.58]. Consumption of any of animal-source foods was higher in the intervention, 54.8%, as compared to the control group, 38.4%, and the difference was statistically significant; [RR = 2; 95% CI: 1.39–2.87].

The proportions of infants who achieved the WHO dietary adequacy indicators between the study groups are presented in Table 6. The proportions of infants who achieved MDD (21% vs. 8%; RR = 3; 95% CI: 1.34–7.39), MMF (62% vs. 39%; RR = 2.4; 95% CI: 1.37–4.29), and the composite indicator MAD (16% vs. 5.6%; RR = 2.7; 95% CI: 1.13–7.23), were significantly higher in the intervention as compared to control groups, respectively.

**Table 6:** Generalized estimated equation regression analyses on the proportion of infants who achieved the dietary adequacy indicators at endline by study groups in rural communities of West Gojjam Zone, Northwest Ethiopia, 2017–2018.

Food items	Study groups	n (%)	*RR (95% CI)
MDD	CG	20 (8)	1
	IG	55 (21)	3 (1.339–7.393)
MMF	CG	97 (39)	1
	IG	158 (62)	2.4 (1.369–4.292)
MAD	CG	14 (5.6)	1
	IG	41 (16)	2.7 (1.131–7.229)

CG: control group (N = 250); IG: intervention group (N = 259); MDD: minimum dietary diversity; MMF: minimum meal frequency; MAD: minimum acceptable diet; RR: relative risk; CI: confidence interval.

*RRs were as yielded by the “Generalized estimated equation regression analyses.”

## Discussion

Complementary foods, particularly in developing countries, are inadequate both in quality and quantity which lack a variety of essential nutrients for optimal growth and development of infants [40]. It is critical to design behavior change intervention strategies that can improve the dietary adequacy of complementary foods for infants.

This study was a community-based cluster-randomized controlled trial aimed to investigate the effectiveness of complementary feeding behavior change communication delivered through community-level actors on the dietary adequacy of infants. The main focus of the intervention was to promote the use of a variety of locally available and affordable nutritious foods to improve the adequacy of traditional home-made complementary diets. The intervention had significantly influenced both the adequacy (both quality and quantity) of complementary diets consumed by children. This suggests that behavior change intervention on optimal complementary food without food supplements can improve dietary diversity and adequacy.

Inappropriate complementary feeding practices with the associated adverse nutrition and health effects in infants remain a significant public health problem worldwide, especially in developing countries [11]. The dietary adequacy of an infant’s food is dependent on meal frequency and food groups contained in the diet [1]. Improving the quantity and quality of infant’s food in this critical windows period is among the most cost-effective strategies to improve overall health and ensure nutritional wellbeing [42].

In this study, the intervention had positive statistically significant effects on the consumption of dairy products, eggs, vitamin A-rich fruits and vegetables; other fruits and vegetables; and animal-source foods. The consumptions of dairy products (53.3% vs. 37.6%; RR = 1.8; 95% CI: 1.04–3.13), eggs (25.5% vs. 9.6%; RR = 3; 95% CI: 1.35–6.56), vitamin A-rich fruits and vegetables (23% vs. 9.2%; RR = 2.7; 95% CI: 1.17–6.1), and other fruits and vegetables (32.4% vs. 14.4%; RR = 5; 95% CI: 2.49–10.58) were significantly higher in infants in the intervention as compared to those in the control groups, respectively. Similarly, the proportion of infants who consumed any of the animal-source foods was significantly higher in the intervention group, 54.8%, as compared to the control group, 38.4% (RR = 2; 95% CI: 1.39–2.87).

Complementary foods mostly are plant-based which lack the essential nutrients for growth and development of children 6–24 months of age, particularly in developing countries. It is therefore recommended to feed children a variety of animal-source foods (flesh meats, eggs, and dairy products) since they are rich in protein and micronutrients which are important for optimal health and nutrition.

The consumption of animal-source foods can also improve dietary adequacy in infants [43]. In our study, the significant effects of the intervention on the consumption of eggs and dairy products could be due to poultry and cow rearing are common practices in the study area that would make eggs and dairy products accessible in the intervention clusters. Nonetheless, the intervention had not shown a significant difference in the proportion of infants that consumed meats. This could be explained by meats are relatively expensive in the study area and might not be affordable for most households regularly.

The intervention had positive significant effects on the proportion of infants who achieved the WHO dietary adequacy indicators. The proportions of infants who achieved the MDD (21% vs. 8%; RR = 3; 95% CI: 1.34–7.39), MMF (62% vs. 39%; RR = 2.4; 95% CI: 1.37–4.29), and MAD (16% vs. 5.6%; RR = 2.7; 95% CI: 1.13–7.23) were significantly higher in the intervention than control groups, respectively. This was due to the achievement of higher consumption of frequencies of food groups in the intervention than control groups.

The result of this trial is supported by the findings of studies conducted in different parts of the world. A cluster-randomized controlled trial conducted in Malawi applied group training and individual counseling to promote optimal complementary feeding practices mainly targeting caregivers of children. The nutrition education intervention was designed as a series of ten facilitated sessions. Pairs of trained volunteers facilitated the sessions in their home villages. The sessions covered topics on the selection of age-appropriate food, nutrients, diet, feeding children, food preparation, water, sanitation, and hygiene. The intervention showed a significant positive effect on MDD and MAD but not on MMF [44].

In another behavior change intervention carried out in India, health and nutrition workers in the intervention communities conducted counseling on complementary feeding for caregivers based on locally developed feeding recommendations through monthly home visits. The meal frequencies and energy intakes were significantly higher in the intervention communities but the intervention failed to improve significantly the proportion of infants who achieved MDD and MAD between the control and intervention groups [45].

A community-based educational intervention was conducted in Kenya. This nutrition education intervention consisted of four sessions comprising of group training and cooking demonstrations that were conducted over a period of 5 months. In this trial, significantly higher proportions of children achieved the MDD, MMF, and MAD in the intervention group compared with the control group at endline. The consumption of frequencies different food groups also improved significantly among children in the intervention group after the nutrition education program but failed to significantly improve the consumption of animal-source foods [46].

The trials discussed above were community-based behavior change interventions without the provision of food and the key messages were also focused on the use of locally available foods like our study. However, many of the interventions targeted only mothers/caregivers and did not engage the family members. This could be the reason why some of the interventions failed to improve either the quality or quantity of the complementary foods or consumption of animal-source foods.

In our study, the behavior change intervention significantly improved the dietary adequacy (both the quality and quantity) of complementary foods including the consumption of the proportion of animal-source foods. This could be due to different reasons. First, our intervention was delivered through community-level actors who would have influence change in traditional norms about feeding behavior and encourage the adoption of the recommended feeding practices in the community. Second, the intervention targeted not only mothers but also family members (fathers and grandmothers of recruited infants) that would provide a supportive environment for behavior change to mothers. The mothers were also taught how to incorporate locally available and affordable nutrition foods into the existing home-made complementary foods during cooking demonstrations that can be well accepted by mothers/caregivers.

Community-based nutrition education for behavior change has the potential to improve complementary feeding practices. Through raised awareness and knowledge, changes in behavior can be expected, thereby improving the adequacy of complementary diets [47]. However, nutrition education alone may not be sufficient in improving complementary feeding practices particularly in countries with low agricultural productivity, and with poverty. This is due to the fact that low agricultural productivity and poverty can directly affect the availability, affordability and utilization of food in the caregiver’s household. Improved agricultural production and/or purchasing power can increase access and consumption of nutritious foods, and result in improving the adequacy of complementary diet [48].

### Strength and limitation of the study

The strengths of this study are the use of the randomized controlled design that included a fairly large sample size. Because the intervention was delivered through community-level actors, it improved the chance of sustainability. Our study has some limitations. First, due to the nature of the study, it was not possible to conduct a double-blind trial. Second, dietary adequacy measurements depend on maternal dietary recall and were not based on direct observations. Hence, recall bias cannot be excluded even if data collectors applied various probing techniques. Third, dietary adequacy was measured using self-reported data. These data could be affected by social-desirability bias, which might have led to the over-reporting of the desirable practices. Fourth, our study had only two data collection sessions (at baseline and endline). We recommend that similar studies in the future be accompanied with more follow-up visits and assessments to reinforce behavior change and adoption of the recommended infant feeding practices.

## Conclusion

This study indicates the potential effectiveness of complementary feeding behavior change communication delivered through community-level actors in improving the dietary adequacy of infants. The results suggest that behavior change intervention that engaged not only mothers of infants but also their family members could be an effective approach. Significant changes can also be achieved on dietary adequacy through the promotion of a variety of locally available and affordable nutritious foods that can be well accepted by mothers.

## Supporting information

S1 Checklist (DOC)

S1 Questionnaire Questionnaire (English version). (DOCX)

S2 Questionnaire Questionnaire (Amharic version). (DOCX)

S1 Protocol (DOCX)

S1 Data (SAV)

S2 Data (XLSX)
