# Supplementary material for: Effect of complementary feeding behavior change communication delivered through community-level actors on dietary adequacy of infants in rural communities of West Gojjam Zone, Northwest Ethiopia: A cluster-randomized controlled trial
Source: PLoS One. 2020 Sep 3;15(9):e0238355. doi: 10.1371/journal.pone.0238355 (PMC7470293; doi:10.1371/journal.pone.0238355)
Supplement: S1 Questionnaire — (DOCX) [file pone.0238355.s002.docx]

**Part-I: Baseline data collection questionnaire, 2017.**

**Administrative details**

| Data collection date: ____/___/_____ | District Name: | Cluster Name: |
| --- | --- | --- |
| Cluster No: | Household No: | Questionnaire Id.No: |
| Data collectors’ Name & signature: | Supervisors’ Name & signature: | Visit completed:  1 Yes  2 No (specify the reason):…………… |

**Section-A: Household socio-demographic data**

| **NO.** | **Questions & filters** | **Responses & coding categories** |
| --- | --- | --- |
| A1 | How old are you? | …………..years |
| A2 | What is your current marital status | 1 Single A6  2 Married  3 Divorced A6  4 Widowed A6 |
| A3 | Did your partner attend any formal school? | 1 Yes  2 No 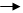A5 |
| A4 | What is the highest level of school your partenr completed? | 1 Primary  2 Secondary  3 Preparatory  4 Technical /Vocational certificate  5 College/University |
| A5 | What is your partner occupation? | 1 Government employee  2 Farmer  3 Merchant/ small scale trading  4 Laborer  5 Other (specify):……………. |
| A6 | Have you ever attended school? | 1 Yes  2 No 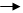 A8 |
| A7 | What is the highest level of school you completed? | 1 Primary  2 Secondary  3 Technical/Vocational certificate  4 College/University |
| A8 | What is your occupation? | 1 Government employee  2 Farmer  3 Merchant/small scale trading  4 Laborer  5 House wife  6 Other (specify):……………. |
| A9 | What is your religion? | 1 Orthodox  2 Catholic  3 Protestant  4 Muslim  5 Others (specify):………….. |
| A10 | How many people usually live together in your household? | …………..peoples |

**Section-B: Maternal obstetric and health service utilization history**

| **No.** | **Questions & filters** | **Responses & coding categories** |
| --- | --- | --- |
| B1 | Sex of the index child | 1 Male  2 Female |
| B2 | Name of the index child | ………………………………... |
| B3 | Age of the index child? | …………..months |
| B4 | How many pregnancies have you ever had? | …………...pregnancies |
| B5 | What is the birth order of *[Name]*? | …………… |
| B6 | How many times did you receive ANC during this pregnancy? | ………….. |
| B7 | Where did you give birth to *[Name]*? | 1 Home  2 Health facility |
| B8 | When [Name] was born, how was of his/her weight? | 1 Large  2 Medium  3 Small |
| B9 | After you gave birth to *[Name],* did anyone check your health? | 1 Yes  2 No |
| B10 | Did you have discussion about IYCF with HEWs or health professionals during this pregnancy or after delivery? | 1 Yes  2 No |
| B11 | Does this household currently have a functioning radio? | 1 Yes  2 No **Section-C** |
| B12 | Did you listen a radio drama about IYCF known as “seven solutions”? | 1 Yes  2 No |

**Section-C: Maternal attitude about complementary feeding practices**

*Now I would like to ask you about your opinion about* complementary feeding practices *and please tell me if you agree, disagree or do not know.*

| **No** | **Statements** | **Responses**  **1 Agree**  **2 Disagree**  **8 Do not know (DNK)** |
| --- | --- | --- |
| C1 | Breastfeeding alone is not sufficient for a child after 6 months |  |
| C2 | Giving complementary foods after 6 months makes a baby healthy |  |
| C3 | It is good to give baby fruits and vegetables |  |
| C4 | A baby needs animal source foods |  |
| C5 | Bottle feeding is not good for child’s health |  |
| C6 | Giving extra meal is desirable before and after an illness |  |

**Section-D: Maternal knowledge on complementary feeding practices**

*Now I would like to ask you some questions about* complementary feeding practices*.*

| **No.** | **Questions** | **Responses** |
| --- | --- | --- |
| D1 | For how long in months your baby can survive and grow on breast milk alone even without water? | 1……….months  8 DNK |
| D2 | When should mothers start to give their baby complementary food? | 1……….months  8 DNK |
| D3 | How many times should a 6-8 months age breastfed baby eat complementary foods each day (24 hours)? | 1.……..no of times  8 DNK |
| D4 | How many times should a 9-23 months age breastfed baby eat semi-solid, solid and soft foods each day (24 hours)? | 1……..no of times  8 DNK |
| D5 | A baby 6-23 months of age require a minimum of 4 food groups | ………….. |
| D6 | Non-breastfed baby needs extra meal | 1 Yes  2 No |

**Part-II: Endline data collection questionnaire, 2018.**

**Administrative details**

| Data collection date: ____/___/_____ | District Name: | Cluster Name: |
| --- | --- | --- |
| Cluster No: | Household No: | Questionnaire Id.No: |
| Data collectors’ Name & signature: | Supervisors’ Name & signature: | Visit completed:  1 Yes  2 No (specify the reason):……… |

**Mother complementary feeding practices for her child**

| **No.** | **Questions & filters** | **Responses & coding categories** |
| --- | --- | --- |
| A1 | Have you started giving for *[Name]* any solid, semi-solid, or soft foods? | 1 Yes  2 No **Stop** |
| A2 | At what age in months did you first give solid or semi-solid food to [Name]? | ………….months |
| A3 | Yesterday during the day or at night, did *[Name]* eat any solid, semi-solid, or soft foods? | 1 Yes  2 No **Stop** |
| A4 | How many times did *[Name]* eat solid, semi-solid or soft foods yesterday during the day or at night? | 1…………..no of times  8 DNK |
| A5 | **24 hour diet recall:** Please describe the foods your child ate yesterday during the day and night (from sunrise yesterday to sunrise today) in your home. *[Write down all food and drinks mentioned by the respondent. When the respondent has finished, probe for meals and snacks not mentioned. Once you listed the foods and drinks, tick the food items consumed mentioned in the following table].* | |

**Food items consumed**

| **No** | **Food groups** | **Questions and filters** | **1 Yes**  **0 No** |
| --- | --- | --- | --- |
| 1 | Grains, roots & tubers | *Injera*, bread, rice, noodles, or other foods made from grains, such as, teff, oats, maize, barley, wheat, sorghum, millet or other grains, white potatoes, white yams, bulla, kocho, manioc, cassava or any other foods made from roots? |  |
| 2 | Legumes & nuts | Any foods made from beans, peas, lentils, nuts? |  |
| 3 | Dairy products | Cheese, yogurt, or other milk products? |  |
| 4 | Flesh food | Any meat such as beef, pork, lamb, goat, chicken, or fish? |  |
| 5 | Eggs | Egg? |  |
| 6 | Vitamin A-rich fruits and vegetables | Pumpkin, carrots, sweet potatoes? |  |
| 7 | Other fruits and vegetables? | Any other vegetables & fruits |  |
