# Supplementary material for: Effect of complementary feeding behavior change communication delivered through community-level actors on dietary adequacy of infants in rural communities of West Gojjam Zone, Northwest Ethiopia: A cluster-randomized controlled trial
Source: PLoS One. 2020 Sep 3;15(9):e0238355. doi: 10.1371/journal.pone.0238355 (PMC7470293; doi:10.1371/journal.pone.0238355)
Supplement: S2 Questionnaire — (DOCX) [file pone.0238355.s003.docx]

ክፍል-1፡ የመጀመርያ ዙር የመረጃ የመሰብሰብያ ቅፅ፣ 2009 ዓ.ም

አስተዳደራዊ ዝርዝር

| የጉብኝት ቀን፡ ______/______/______ | የወረዳ ስም፡ | የጎጥ ስም፡ |
| --- | --- | --- |
| የክለስተር ቁጥር፡ | የቤት ቁጥር፡ | መለያ ቁጥር፡ |
| መረጃ ሰብሳቢ ስምና ፊርማ፡ | የሱፐርቫይዘር ስምና ፊርማ፡ | መረጃ መሰብሰቡ ተሳከቷል?  1 አዎ  2 የለም (ምክንያቱ ይገለፅ)፡……… |

ክፍል-A: አጠቃላይ መረጃ

| ቁጥር | ጥያቄዎችና ማጣርያዎች | መልስና መለያ ቁጥር |
| --- | --- | --- |
| A1 | እድሜሽ ስንት ነዉ | ………………ዓመት |
| A2 | በአሁኑ ሰአት የትዳር ሁኔታሽ | 1 ብችየን A6  2 ባለትዳር  3 አግብታ የፈታች A6  4 ባሏ የሞተባት A6 |
| A3 | ባልሽ የተማረ ነዉ? | 1 አዎ  2 አይደለም A5 |
| A4 | ባልሽ እስከ ስንት ተምሯል? | 1 መጀመርያ ደረጃ  2 ሁለተኛ ደረጃ  3 መሰናዶ  4 ሙያና ቴክኒክ/ሰርትፊኬት/  5 ኮሌጅ/ዩኒቨርስቲ |
| A5 | የባልሽ ስራ ምንድን ነዉ? | 1 የመንግስት ተቀጣሪ  2 ገበሬ  3 ነጋዴ  4 የቀን ሰራተኛ  5 ሌላ ካለ ይጠቀስ |
| A6 | አንች ተምረሻል? | 1 አዎ  2 የለም A8 |
| A7 | እስከ ስንት ተምረሻል? | 1 መጀመርያ ደረጃ  2 ሁለተኛ ደረጃ  3 ሙያና ቴክኒክ/ሰርትፊኬት/  4 ኮሌጅ/ዩኒቨርስቲ |
| A8 | ስራሽ ምንድን ነዉ? | 1 የመንግስት ተቀጣሪ  2 ገበሬ  3 ነጋዴ  4 የቀን ሰራተኛ  5 የቤት እመቤት  6 ሌላ ካለ ይጠቀስ፡.................... |
| A9 | ሀይማኖትሽ ምንድን ነዉ? | 1 ኦርቶዶክስ  2 ካቶሊክ  3 ፕሮቴስታንት  4 እስልምና  5 ሌላ ካለ ይጠቀስ፡...................... |
| A10 | የቤተሰብ ብዛት? | ………………….ሰዎች |

ክፍል-B: የእናትየዋ የእርግዝናና ጤና አገልግሎት አጠቃቀም በተመለከተ

| ቁጥር | ጥያቄዎችና ማጣርያዎች | መልስና መለያ ቁጥር |
| --- | --- | --- |
| B1 | የልጁ ጾታ | 1 ወንድ  2 ሴት |
| B2 | የልጁ ስም | ………………………………. |
| B3 | የልጁ እድሜ | …………………..ወራት |
| B4 | ከአሁኑ ጋር ስንቴ አርግዘሻል | ………………….. |
| B5 | የአሁኑ ስንተኛ ልጅሽ ነዉ | …………………… |
| B6 | የአሁኑን እርግዝና ክትትል ስንት ግዜ አድርገሻል | …………………… |
| B7 | የአሁኑ ልጅሽን የወለድሽዉ የት ነዉ | 1 ቤት ዉሰጥ  2 የጤና ተቀም |
| B8 | በአንቺ ግምት ልጅሽ ሲወለድ መጠኑ ምን ነበር? | 1 ትልቅ  2 መካከለኛ  3 ትንሽ |
| B9 | ከወለድሽ በሃላ የጤናሽን ሁኔታ ያየሽ አለ? | 1 አዎ  2 የለም |
| B10 | በአሁኑ እርግዝናሽ ወይም ከወለድሽ በሃላ ስለጨቅላ አመጋገብ ከጤና ባለሙያ ወይም ከህብረተሰብ ጤና ሰራተኛ ጋር ዉይይት አድርገሽ ታዉቂያለሽ? | 1 አዎ  2 የለም |
| B11 | ቤት ዉስጥ በአሁኑ ሰአት የሚሰራ ራዲዎ አለ | 1 አዎ  2 የለም ምድብ-**C** |
| B12 | ስለጨቅላ አመጋገብ ህጻናት የሚያተኩር “ሰባት መላ” የሚባል የራዲዎ ድራማ ታደምጫለሽ | 1 አዎ  2 የለም |

ክፍል-**C**: እናት ስለህጻን ልጅ አመጋገብ ያላትን አመለካከት በተመለከተ

አሁን ስለጨቅላ ህጻናት አመጋገብ ያለሽን አመለካከት ስለምጠይቅሽ እስማማለሁ ወይም አልስማማም በማለት መልሽልኝ

| ቁጥር | ዓረፍተ-ነገር | መልስ  1 እስማማለሁ  2 አልስማማም  8 አላዉቀዉም |
| --- | --- | --- |
| C1 | ከ6 ወር በኋላ ለልጁ የእናት ጡት ወተት ብቻ በቂ አይደለም |  |
| C2 | ለህፃን ልጅ ከ6 ወር በኋላ ተጨማሪ ምግብ መስጠት ጠናማ ያደርገዋል |  |
| C3 | ለልጅ ፍራፍሬና ቅጠላቅጠል መመገብ ጠቃሚ ነዉ |  |
| C4 | ለልጅ የእንስሳት የምግብ ተዋጽኦ መመገብ ጠቃሚ ነዉ |  |
| C5 | በጠርሙስ ወይም በብልቃጥ መመገብ ለልጅ ጠና ጥሩ አይደለም |  |
| E6 | ለልጅ በታመመ ግዜና በኋላ ተጨማሪ ምግብ መስጠት ጠቃሚ ነዉ |  |

ክፍል-D: እናት ስለህፃን ልጅ ህጻናት አመጋገብ ያላትን እዉቀት በተመለከተ

አሁን ስለጨቅላ ህጻናት አመጋገብ በተመለከተ አንዳንድ ጥያቄወች እጠይቅሻለሁ

| ቁጥር | ጥያቄ | መልስ |
| --- | --- | --- |
| D1 | ልጅ በእናት ጡት ወተት ብቻ ያለምግብና መጠጥ ዉሃንም ጨምሮ ሳይሰጠዉ ለስንት ግዜ ያህል መኖርና ማደግ ይችላል? | 1................ወር  8 አላዉቅም |
| D2 | እናት ለልጃ መቼ ነዉ ተጨማሪ ምግብ መስጠት መጀመር ያለባት | 1................ወር  8 አላዉቅም |
| D3 | ከ6 እስከ 8 ወር እድሜ ያለዉ ጡት የሚጠባ ልጅ በቀን ዉስጥ ስንቴ ተጨማሪ ምግብ መመገብ አለበት | ........... |
| D4 | ከ9 እስከ 23 ወር እድሜ ያለዉ ጡት የሚጠባ ልጅ በቀን ዉስጥ ስንቴ ተጨማሪ ምግብ መመገብ አለበት | ............ |
| D5 | እድሜዉ ከ6-23 ወር የሆነ ልጅ ስንት አይነት የምግብ አይነቶች ያስፈልጉታል | ........... |
| D6 | ጡት ያልጠባ ልጅ ከጠባ ልጅ በላይ ተጨማሪ ምግብ ያስፈልገዋል | 1 አዎ  2 የለም |

ክፍል-2፡ የሁለተኛ ዙር የመረጃ መሰብሰቢያ ቅፅ፣ 2010 ዓ.ም

| የጉብኝት ቀን፡ ______/______/______ | የወረዳ ስም፡ | የጎጥ ስም፡ |
| --- | --- | --- |
| የክለስተር ቁጥር፡ | የቤት ቁጥር፡ | መለያ ቁጥር፡ |
| መረጃ ሰብሳቢ ስምና ፊርማ፡ | የሱፐርባዘር ስምና ፊርማ፡ | መረጃ መሰብሰቡ ተሳከቷል?  1= አዎ  2= የለም (ምክንያቱ ይገለፅ)፡……… |

እናት ለልጇ ያላት የአመጋገብ ልምድ

| ተራ  ቁጥር | ጥያቄዎች | መልስና መለያ ቁጥር |
| --- | --- | --- |
| A1 | ለልጁ ተጨማሬ ምግብ መስጠት ጀምረሻል? | 1 Yes  2 No አቁም |
| A2 | ለመጀመርያ ግዜ ምግብ ስትሰጭዉ እድሜዉ ስንት ነበር? | ………….ወር |
| A3 | ትላንት ቀንም በማታንም ጨምሮ ለልጅሽ ምግብ ሰጥተሸዋል? | 1 Yes  2 No አቁም |
| A4 | ትላንት ቀኑንም ማታንም ጨምሮ ልጅሽ ስንት ግዜ ምግብ ተመግቧል? | 1………..ግዜ  8 አላዉቀዉም |
| A5 | ትላንት ቀኑንም ማታንም ጨምሮ በቤትሽ ዉስጥ ልጅሽ የተመገበዉን የምግብ አይነት ዘርዝሪልኝ፡፡ ልጁ የተመገበዉን መግብና መጠጥ በዝርዝር መልክ ከዚህ ታች አስቀምጥ/ጭ፡፡ | |

የምግብ አይነቶች

| ተራ ቁጥር | የምግብ አይነቶች | ጥያቄዎችና መለያዎች | 1 አዎ  0 የለም |
| --- | --- | --- | --- |
| 1 | ጥራጥሬ እና ስራስሮች | እንጀራ፣ ዳቦ፣ ሩዝ ሌሎች ጥራጥሬዎች (ጤፍ፣ አጃ፣ ማሽላ፣ ገብስ፣ ስንዴ፣ ዘንጋዳ፣ ኩቾ፣ ገያ፣ ሽምብራ)፣ ድንች፣ ቡላ፣ ካሳባ ሌሎች ስረ ገንድ ምግቦች እና ሌሎችም |  |
| 2 | የወጥ እህል ዘሮች | ባቄላ፣ አተር፣ ምስር፣ ኑግ፣ ሱፍ፣ ተልባ |  |
| 3 | ወተት እና የወተት ተዋፅኦ | ጥሬ ወተት፣ እርጎ፣ አይብ እና ሌሎች የወተት ተዋፅወች |  |
| 4 | ስጋ | ስጋ (የከብት፣ የፍየል፣ የበግ፣የዶሮ፣ የአሳማ፣ የጥጃ ሌላም) |  |
| 5 | እንቁላል | እንቁላል |  |
| 6 | በቫይታሚን-ኤ የበለፀጉ ፍራፍሬዎችና ቅጠላቅጠሎች | ማንጎ፣ ፓፓያ፣ ዱባ፣ ካሮት፣ ስካር ድንች |  |
| 7 | ሌሎች ፍራፍሬዎችና ቅጠላቅጠሎች | ሌሎች ቅጠላቅጠሎችና ፍራፍሬዎች |  |
