# Supplementary material for: Effect of complementary feeding behavior change communication delivered through community-level actors on dietary adequacy of infants in rural communities of West Gojjam Zone, Northwest Ethiopia: A cluster-randomized controlled trial
Source: PLoS One. 2020 Sep 3;15(9):e0238355. doi: 10.1371/journal.pone.0238355 (PMC7470293; doi:10.1371/journal.pone.0238355)
Supplement: S1 Protocol — (DOCX) [file pone.0238355.s004.docx]

***Effect of complementary feeding behavior change communication delivered through community-level actors on feeding practices, health, and growth of infants in rural communities of West Gojjam Zone, Northwest Ethiopia: a cluster-randomized controlled trial***

**Chalachew Abiyu^1^**^*^**, Tefera Belachew^2^**

***^1^****School of Medicine****,*** *College of Medicine and Health Sciences, Wollo University, Dessie, Ethiopia.*

***^2^****Faculty of Public Health, Department of Nutrition and Dietetics, Jimma University, Jimma, Ethiopia.*

*Correspondence: [chalachewabiyu@yahoo.com](mailto:chalachewabiyu@yahoo.com)

**Abstract**

**Introduction:** Globally, about 40% of child mortality less than two years is associated with inappropriate feeding practices. Over two-third of malnutrition is associated with inappropriate feeding practices during the first year of life.

Inappropriate infant and young child feeding practices are widely documented in Ethiopia. As a result, the problem of stunting has remained pervasively high in the country. This could be not only due to lack of food but also associated with poor knowledge, harmful cultural norms and behaviors of mothers.

The promotion of optimal complementary feeding through behavior change interventions is a global health priority. However, many of the interventions targeted only mothers/caregivers of infants and studies that engaged other family members are limited worldwide. Moreover, such interventions are scarce in developing countries including Ethiopia. This study aimed to evaluate the effectiveness of complementary feeding behavior change communication delivered through community-level actors on the feeding practices, health and growth of infants.

**Methods:** A cluster randomized controlled trial which will be conducted in rural communities of West Gojjam Zone, Northwest Ethiopia. Complementary feeding behavior change communication will be conducted in the intervention clusters for 8 months whereas the control group will receive only the routine care. The intervention will be delivered by trained women development army leaders. Data will be collected by pre-tested, structured interviewer-administered questionnaires. The data will be checked, coded and double entered using EPI info and exported to SPSS version 21 for statistical analysis. Linear mixed models and generalized estimating equations adjusted for clustering will be used to test the intervention effects for continuous and categorical variables, respectively. Survival analysis will be conducted to compare the time of initiation of complementary foods in the control and intervention groups.

**Significance of the trial:** The result of this trail could be useful for health and nutrition policymakers and other concerned bodies in decision making and to design effective intervention strategies to improve feeding practices, health, and growth of infants.

**Trial registration:** The trial will be registered on ClinicalTrials.gov.

**Keywords: Complementary feeding, Behaviour change communication, Feeding practice, Health, Growth**

**Introduction**

Child undernutrition is a major risk factor for ill-health and mortality, contributes substantially to the burden of disease in low-income and middle-in­come countries (LMICs) and is associated with close to half of all child deaths which include stunting, severe wasting, and micronutrient deficiencies [1]. This is mainly due to its influence on morbidity from the major causes of child deaths including acute respiratory illnesses, diarrhea, malaria, and measles. Undernutrition is also a major factor for loss in disability-adjusted life years (DALYs), causing 81 million (18 %) loss in DALYs in children under 5 years [2]. Furthermore, it is associated with other adverse outcomes including compromised cognitive development, scholarly achievement, and future economic productivity; and a higher risk of metabolic diseases later in the life course [3].

Childhood malnutrition is prevalent in LMICs, and according to an estimate, 19.4% of children <5 years of age in these countries were underweight and about 29.9% were stunted in the year 2011[4]. The prevalence of both underweight and stunting is highest in Africa and South-central Asia [5]. Ethiopia is one of the poorest countries in Sub-Saharan Africa, and child malnutrition is a serious public health problem where the rates for stunting (40%), underweight (25%) and wasting (9%) among children under 5 years are among the highest in the world [6-8].

According to the WHO, inappropriate feeding practices are one of the major causes of high morbidity in young infants in the developing world. Inappropriate feeding practices increase the risk of undernutrition, illness, and mortality in infants and young children less than 2 years of age. Malnourished children who survive also get more frequently sick and suffer from life-long consequences of malnutrition in their life and perhaps the effect will span generations [9, 10].

The first two years of life provide a critical window of opportunity for ensuring appropriate growth and development of children from generation to generation through optimal feeding. Any damage caused during this period can lead to impaired cognitive development, compromised educational achievement, low economic productivity, and malnutrition because the first two years of a child’s life are the most important for establishing healthy growth. Once growth faltering occurs in this age, there is little opportunity for catch-up growth. Stunted infants grow to be stunted children and stunted adults [11].

WHO and UNICEF’s recommendations for optimal infant and young child feeding are early initiation of breastfeeding within one hour of birth, exclusive breastfeeding for six months and nutritionally adequate and safe complementary feeding starting from the age of six months with continued breastfeeding up to two years of age or beyond. WHO also has developed indicators to assess infant and young child feeding practices [12-13]. Complementary feeding for infants refers to the timely introduction of safe and nutritional foods in addition to breastfeeding i.e. clean and nutritionally rich additional foods introduced at about six months of infant age. According to the WHO, Complementary feeding should be timely, adequate, appropriate, and given in sufficient quantity [14, 15].

Adoption of recommended breastfeeding and complementary feeding practices and access to the appropriate quality and quantity of foods are essential conditions for fulfilling optimal nutrition for infants and young children. Globally, about 40% of death children less than two years is associated with inappropriate feeding practices. Optimal breastfeeding and appropriate complementary feeding could prevent 13% and 6% under-five mortality, respectively. Over-two third of malnutrition is associated with inappropriate feeding practices during the first year of life [16, 17].

Every day, 3000-4000 infants die in the developing world from diarrhea and acute respiratory infections because they are given inadequate amounts of breast milk. More than 10 million children die each year in sub- Saharan Africa and South Asia. A major contributor to their deaths is poor breastfeeding practice. The risk of death from diarrhea of partially breastfed infants 0-6 months of age was 8.6 times the risk for exclusively breastfed children [18, 19].

Infant and young feeding practices are not optimal worlwide. It is only 34.8% of infants are exclusively breastfed worldwide. Complementary foods are often introduced too early or too late and are often nutritionally inadequate or unsafe. Only about 39% of infants in developing countries, 25% in Africa are exclusively breastfed for the first six months and 6% of infants in developing countries are never breastfed [20].

A wide range of harmful infant and young child feeding practices were documented in Ethiopia. According to EDHS of 2011, 52% of infants started breastfeeding within one hour of birth and exclusive breastfeeding during the first six months with the 4.2 months mean duration of exclusive breastfeeding. About half (49%) of children aged 6-8 months consumed solid, semi-solid, or soft foods and 5% of children were fed minimum dietary diversity and 4% of children fed minimum meal frequency per day while 96% of children continued breastfeeding at one year, and 82% continued at 2 years. Only 4% of children 6-23 months living with their mothers are fed in line with IYCF practices and 66% of children under the age of two received age-appropriate breastfeeding. Overall, nearly three children in every ten (27%) are given prelacteal feeds within the first three days of life [21].

Suboptimal infant and young child feeding practices are associated with caretakers’ poor knowledge, lack of information and being restricted by traditional beliefs. It is essential to give caregivers the necessary knowledge and information to alter their inappropriate feeding behaviors [22-23]. To sustain the gains made by promoting exclusive breastfeeding for the first six months of life, interventions need to extend into the second half of infancy and beyond. This could be ensured by enabling caregivers to appropriately feed their children with safe and adequate complementary foods while maintaining frequent breastfeeding [24].

Several efforts to improve the feeding status of the infant and young children have been carried out at different times. The Ethiopian government developed the IYCF guideline in 2004 following WHO recommendations of global strategy for feeding infants and young children for proper nutrition & health [25]. However, these efforts have failed to bring about substantive and sustainable changes leading to the improvement of IYCF practices since efforts (nutrition actions) were not based on the evidence on existing feeding practices and what works and what does not. As a result, the problem of stunting has remained pervasively high in the country [26].

In Ethiopia, a few behavior change interventions aimed at improving the IYCF practices have been conducted by the Non-governmental organizations (NGOs) projects [27-30]. The reports of these projects focus either on implementation fidelity [27], or are implementation research [28], and large scale in scope, focusing not only on complementary feeding but also on other IYCF practices [29-31]. Moreover, none of the interventions targeted on age-specific complementary feeding practices, engaged community-level actors and delivered before infants entered the complementary feeding period (before 6 months). None of the projects also used control groups except a trial conducted in Hula woreda, Southern Ethiopia [30]. This study aims to evaluate the effectiveness of complementary feeding behavior change communication delivered through community-level actors in improving feeding practice, health, and growth of infants.

**Conceptual framework**

Increased nutrients intake

Improved growth

Improved health status

Maternal intentions & decision (plan/expect to apply recommendation)

Attitude on complementary feeding

Optimal complementary feeding (frequency, amount, diversity, density, hygiene, and responsive feeding)

Knowledge of complementary feeding

Self-efficacy

Mother’s/Caregiver’s

Social influences

Perceptions of child illness and healthcare-seeking behavior

Health Development army leaders

Figure 1: Conceptual framework illustrating the effect of the intervention in improving feeding practice of mother, nutrition and health status of infants. ***Source:*** Developed by reviewing different literature.

**Objective and Hypothesis**

The primary objective of the intervention trial is to evaluate the effectiveness of community feeding behavior change communication delivered through community-level actors in improving feeding practices, health, and growth of infants. We hypothesize that the community feeding behavior change communication delivered through community-level actors will be more effective than the routine/usual health services. The primary outcome will be feeding practices, health and growth. Secondary outcomes will include the level of maternal knowledge and attitude on the recommended complementary feeding practices, and health-seeking behavior. The information generated from this trial could be useful for health and nutrition policymakers and other concerned bodies in decision making and to design appropriate and effective intervention strategies to improve feeding practices with the view of mitigating child malnutrition and improving their growth and health.

**Methods**

**Study setting**

This trial will be conducted in rural communities of West Gojjam Zone, Ethiopia from February 2017 to March 2018. West Gojjam Zone is one of the 13 administrative zones of the Amhara regional state. It has 13 rural districts*,* and each district is divided into *kebeles,* the lowest administrative units in Ethiopia*.* According to the population projection of Ethiopia for all regions at the district level from 2014 to 2017, which is based on the 2007 national census, the zone has a total population of 2,560,131in 2016; of whom 1,262,144 were male and 1,297,987 were female. The rural part accounts for 92% of the total population. A total of 480,255 households were counted in this Zone, which results in an average of 4.39 persons to a household, and 466,491 housing units. From the total population mentioned, 315,228 were children of under five years of age of whom 160, 214 were under two years of age [32].

In the Amhara region, a total of 117,428 Health Development Army (HDA) groups and 532,259 one-to-five networks were established in 2011 [33]. The one-to-five networks are women volunteers who are empowered as an HDA to transform their society. They are trained to focus more intensively on sparking local behavior change making regular rounds to check on neighbors and encourage healthy lifestyles. They are from “model families” and serve as living examples that the health extension workers (HEW) messages are being heard [34]. The proportion of women of childbearing age is 24% [35].

**The context**

The Ethiopian government started the Health Development Army (HDA) in 2011intending to consolidate the gains made by the health extension programs (HEP) and promote community ownership of the programs. Although some regions have both male and female HDAs, HDAs are now basically women known as the women development army (WDA) [35].

WDAs are selected from the model families. Once the WDA groups are formed through participatory community involvement, the WDA leaders provided an intensive 7 to 10 days training [35], whose primary objective is to educate and mobilize the communities to utilize the maternal, neonatal and child health (MNCH) services delivered by the health post and health centers [36]. (24). In average, there are approximately 30 WDA team leaders and 200 WDA network leaders in each *kebele* [35].

Each WDA group comprised 25-30 households (women) which are further organized into the “1 to 5” network of women where a model woman leads five other women within her neighborhood [37]. The one-to-five network functions as a forum for the exchange of concerns, priorities, problems, and decisions related to the health status of women. While being supported by the health extension workers (HEW), the networks are responsible for the preparation of plans and ensuring their completion, for the collection of health information, and also for conducting a weekly meeting to review progress and submitting monthly reports [38]. The WDA groups thus support the implementation of the HEP [[Figure 1].](file:///C:\Users\toshi\Desktop\Plos%20M-II\Figures.docx)

The one-to-five networks meet every week, while the larger health development team meets once every two weeks. Moreover, they review their performance against their plan and evaluate each other on a monthly basis and give grades based on their performances. A performance report including the grades is organized at the health development team level and sent to the HEW [35].

In our study context, community-level actors are those people living in the community who could have influence change in harmful traditional feeding behaviors and provide a supportive environment for the adoption of the recommended feeding practices. These include WDA leaders, and family members (fathers and grandmothers).

District Health Office

HEWs

1-5

1-5

1-5

1-5

1-5

WDA leaders

30 households

**Figure 1. Hierarchy of WDA and reporting**

**Study design and population**

A cluster-randomized controlled trial single-blind parallel-group, two-arms trial with a 1:1 allocation ratio will be implemented among mothers of infants aged <6 months of age at the time of enrolment. The trial will be conducted in line with the CONSORT recommendations for cluster-randomized trials [39]. [Figure 2].

Select 2 out of the 13 rural districts of West Gojjam zone by simple random sampling

Screening

Select 16 clusters out of the selected districts by simple random sampling

Cluster-randomization of 16 clusters to two study groups

Randomization

Control groups

(8 clusters)

(7 clusters)

Intervention groups

(8 clusters)

(7 clusters)

- Recruit mothers of infants aged <6 months (N=306)
- Baseline data collection

Recruitment

- Recruit mothers of infants aged <6 months (N=306)
- Baseline data collection

Intervention

Complementary feeding behavior change communication for 8 months

The routine/ usual health services only

Endline

Endline data collection

Endline data collection

**Figure 2: Trial profile**

**Sample size determination**

The sample size is calculated using *G-power* based on the following assumptions (t-test: the difference between two independent means). The effect size of the intervention on infants’ linear growth was considered (the variable which gives the maximum sample size) [40].

- Tail (s): One
- Effect size d: 0.3
- α error probability= 0.05
- power (1- β error probability)= 0.08
- Allocation ratio (N1/N2)= 1

This gives a sample size of 278. Then, it is multiplied by design effect (DE) of 2 and allowing for a 10% loss to follow up a total sample size of 612 will be required (N1= 306 & N2= 306). N1 and N2 are sample sizes in the control and intervention groups, respectively.

**Randomization and Sampling technique**

Because the intervention will be delivered in community settings and encourage collective participation at household settings and facilitate logistical convenience in delivery, the unit of randomization will be the *kebeles* (clusters) in the districts. Each cluster in the district will form a unit of randomization (cluster) for the trial, while mother-infant pairs within the cluster will form units of observation. A list of all clusters in the district will be compiled, and clusters found to have an ongoing intervention or project will be excluded. To minimize information contamination of the intervention, kebeles that did not share geographical boundaries (block randomization) will be selected and listed alphabetically. Then, simple randomization of clusters into the intervention and control groups with a 1:1 allocation will be done using computer-generated random numbers. The generation of allocation sequence and randomization of clusters will be done by a statistician blinded to study groups and not participating in the research. After cluster randomization, the number of mothers of infants aged <6 months in the selected clusters will be obtained from the records of births prepared by HEW. The sample size to be obtained from each cluster will be allocated proportionally to the number of study subjects in each cluster. Then, a simple random sampling method will be used to recruit study subjects from each cluster until the desired sample size is achieved.

**The intervention**

Complementary feeding behavior change communication will be delivered for only intervention clusters for 8 months whereas the control clusters will receive only the routine/usual health care. The language of communication during the intervention delivery will be Amharic (the local language). The intervention will have three parts.

**Part 1: Training of health development army (HDA) leaders**

WDA leaders in each intervention cluster will be recruited by HEW and centrally trained by the researcher. The purpose of the training will be to empower WDA leaders, which acts as community-based IYCF counselors and support groups, with action-oriented knowledge, attitude, and behavior to effectively counsel, support and negotiate with mothers of infants and families to adopt recommended IYCF and health care practices. The training will be provided for two sessions. The first training session will be conducted at the beginning of the intervention whereas the second session of similar content will be repeated 4 months later of the first session. Each session is expected to last 3 days. The training contents will be adopted from the Alive and Thrive IYCF program in Ethiopia [41].

The intervention key messages will focus on the right time to introduce complementary foods; specific foods to be offered or avoided and how to offer them; meal frequencies; amounts of foods to be fed to infants at different ages while continuing breastfeeding; offering a variety of foods from different food groups; practice responsive feeding; practice good hygiene, and continue to feed the child during and after an illness. Direct, interactive (discussion, sharing) and experiential (learner-centered, activity-oriented) instructional strategies will guide the approach of delivering education sessions. Talks, group discussions, group work exercises, demonstrations, role plays, storytelling, simulation, case studies and problem-solving will be applied. Complementary food cooking demonstrations will be done to show procedures for preparing nutritious meals during the sessions [Table-1].

**Table 1: Complementary feeding practices key recommendations and messages in the intervention clusters.**

| **No.** | **Key messages** |
| --- | --- |
| 1 | Start feeding your baby soft and thick porridge made from a combination of cereal flours at 6 months. Continue to breastfeed your child up to 2 years and beyond. |
| 2 | Enrich baby’s porridge by adding one or more ingredients from animal-source foods (milk, egg, dried meat powder), finely chopped vegetables (kale, carrot, cabbage) and mashed fruits (avocado, papaya, mango, banana, pumpkin, tomato, potato) in each meal. |
| 3 | Give undiluted cow’s milk to your child at least 3 times per week. Cook and feed animal-source foods (e.g. eggs, beef, pork, chicken, liver, fish) at least 3 times per week. Feed your child a fruit (e.g. ripe banana, mango, orange, papaya, avocado) after a meal at least once per day. |
| 4 | Increase variety, amount and frequency of feeding meals per day for the baby (2-3 times at 6-8 months, 3-4 times at 9-23 months). Feed 1-2 snacks (e.g. sliced bread, fruits) between two major meals. |
| 5 | Encourage your child to eat with patience and love. Interact and minimize distractions during feeding. Don’t force your baby to eat. Help your older child eat. Provide extra food during and after an illness. |
| 6 | Feed your baby using a clean cup and spoon; avoid bottle feeding. Wash your hands with soap and water before preparing food, before eating, and before feeding young children. |
| 7 | Enriched baby’s porridge preparation:   - Prepare a germinated flour made up of 3/4^th^ staples (one or more ingredients from maize, wheat, rice, millet, sorghum, oat) and 1/4^th^ legumes (one or more ingredients from beans, lentils, chickpeas, groundnuts). - Use milk instead of water for preparing porridge. - Add butter/oil which will make the thick porridge easier to eat. - Add finely chopped meat, fish or eggs. - Add one or more ingredients from finely chopped vegetables and mashed fruits. - Increase the consistency and thickness of the porridge with child age. - Do not forget to use iodized salt. |

**Part 2: Group training of mothers by WDA leaders**

Each member of the trained WDA leader will be assigned to 10-15 mothers with children aged less than 6 months residing in their cluster/village. WDA leaders will deliver eight group training sessions including cooking demonstrations (once per month, for 3 days duration each) for the mothers they are assigned with the same training procedures that will be provided by the researcher in part 1. WDA leaders will use language and culturally appropriate training sessions with mothers using posters.

**Part 3: Home visits**

Each WDA leader will conduct eight home visits (once per month, for 2 days duration each) in the intervention clusters that aimed to bring behavior change at maternal and family level. During each home visit, individual counseling and support will be offered for each mother to reinforce the adoption of feeding practices she had been taught during the group training sessions, feeding practices will be observed, and cooking procedures will be demonstrated. A participatory discussion will be held with family members (fathers and grandmothers of the recruited infant) regarding optimal complementary feeding practice, its impact on children’s nutrition and health; and how can they support the mother in feeding the baby. Each mother will provide a poster containing the key messages at the end of each home visit. The whole activities of WDA leaders will be supervised and monitored by HEW and the overall supervision and monitoring will be done by the researcher. The whole trial work plan is presented in Table 2.

**Table 2: The trial work plan**

| **Activities and measurements** | **Time points in months** | | | | | | | | | |
| --- | --- | --- | --- | --- | --- | --- | --- | --- | --- | --- |
|  | **1** | **2** | **3** | **4** | **5** | **6** | **7** | **8** | **9** | **10** |
| Enrollment and baseline data collection | x**^I+C^** |  |  |  |  |  |  |  |  |  |
| Training of WDA leaders | x**^I^** |  |  |  | x**^I^** |  |  |  |  |  |
| Group training of mothers |  | x**^I^** | x**^I^** | x**^I^** | x**^I^** | x**^I^** | x**^I^** | x**^I^** | x**^I^** |  |
| Home visits |  | x**^I^** | x**^I^** | x**^I^** | x**^I^** | x**^I^** | x**^I^** | x**^I^** | x**^I^** |  |
| Process evaluation |  | x**^I^** | x**^I^** | x**^I^** | x**^I^** | x**^I^** | x**^I^** | x**^I^** | x**^I^** |  |
| Supervision | x**^I+C^** | x**^I^** | x**^I^** | x**^I^** | x**^I^** | x**^I^** | x**^I^** | x**^I^** | x**^I^** | x**^I+C^** |
| Endline data collection |  |  |  |  |  |  |  |  |  | x**^I+C^** |

**^I^**Intervention groups; **^C^**Control groups; **^I+C^**Activities both in intervention and control groups

**Blinding**

Data collectors will not be informed of the allocation clusters and will not be residents in any of the clusters. Due to the nature of the intervention, it will be difficult to mask the intervention allocation for the trial participants.

**Process evaluation**

Process evaluation will be conducted to document the intervention implementation process and to assess whether the intervention activities are implemented as planned, evaluate the performance of WDA leaders and the extent to which the intervention reaches the intended mothers and family members [[Table-3].](file:///C:\Users\toshi\Desktop\Plos%20M-II\Tables.docx)

**Table 3: Process evaluation**

| **Data sources** | **Process indicators** | **Characteristics** |
| --- | --- | --- |
| 1. **Assess whether the intervention activities are implemented as planned** | | |
| Activity logs | - Number of training sessions including cooking demonstrations held with WDA leaders - Number of visual materials distributed to WDA leaders - Number of training sessions including cooking demonstration held with mothers - Number of visual materials distributed to mothers | Fidelity |
| 1. **Evaluate the performance of HDA leaders** | | |
| Attendance records | - Number of recruited WDA leaders - Number of WDA leaders trained - Number of home visits conducted by WDA leaders | Dose delivered (exposure) |
| 1. **Evaluate the extent to which the intervention reached the intended mothers and family members** | | |
| Attendance records | - Number of recruited mother-infant pairs - Number of mothers trained - Number of mothers attended home visits - Number of family members attended home visits | Dose delivered (exposure) |

**Eligibility criteria**

**Inclusion criteria**

All consented mothers of infants aged <6 months at the time of baseline survey, with singleton birth, who are residents in the sampled clusters for at least 6 months and have no plans to move away during the study period will be recruited for the study.

**Exclusion criteria**

Mothers who are ill and unable to communicate and infants with major birth defects, and ill at the time of the baseline survey will be excluded from the study.

**Variables**

**Mian independent variable**

- Complementary feeding behavior change communication

**Main dependent variables**

- Mother’s complementary feeding practices
- Infant’s health status
- Infant’s growth

**Data collection methods and outcome measurements**

Data will be collected using a pre-tested structured interviewer-administered questionnaire. This will include:

- Assessment of the child, maternal, and household characteristics; maternal knowledge and attitude towards the recommended complementary feeding practices using the WHO guideline [42]. Mothers will receive a score of 1 for each correct response and 0 for the wrong response. The scores will be summed to generate an overall knowledge and attitude score, which will be used in further analyses **(at baseline in both study groups)**.
- Complemntary feeding practices of mothers will be assessed according to the key indicators recommended by WHO. The key complementary feeding indicators; time of initiation of complementary foods, minimum dietary diversity (MDD), minimum meal frequency (MMF) and minimum acceptable diet (MAD), will be determined [42]. The dietary intake of children will be determined based on the interactive 24 hours dietary recall. The seven food groups used for determination of these indicators will be: (i) grains, roots & tubers; (ii) legumes and nuts; (iii) dairy products; (iv) flesh foods (meat, poultry, and fish) (v) eggs; (vi) vitamin A-rich fruits and vegetables; and (vii) other fruits & vegetables. Each of the 7 food groups was allocated a score of 1 **(at endline in both study groups).**
- Infant morbidity and maternal healthcare-seeking behavior within two weeks before the visit will be assessed through a maternal recall of the prevalent illness of signs and symptoms **(at endline in both study groups).**
- Anthropometry: recumbent length will be measured to the nearest 0.1 cm using a portable wooden infant/child length board with a fixed head and sliding foot piece. Infants will be weighed with light clothing to the nearest 10 g using a *Salter* scale. The weighing scale and length board will be placed on a flat surface to ensure correct measurements. Each measurement will be done in duplicate and the mean value calculated. Standardized anthropometric procedures will be observed. Nutritional status indices; height-for-age, weight-for-age, and weight-for-height Z scores will be computed will be determined for each child by comparing the child’s measurements with the reference values of the WHO 2006 child growth standards using ANTHRO software **(at baseline and endline in both study groups).**

**Data quality assurance**

- The questionnaires will be prepared in English, and translated to the local language Amharic and then back to English by experts of the language to keep its consistency.
- Careful selection and training of data collectors and supervisors on the tools and methods of data collection by the principal investigator.
- A Pre-test will be done on 5% of the sample in a community with a similar status to the study community before the actual data collection to check the completeness, consistency, and applicability of the instruments, and will be ratified accordingly.
- Training materials for the intervention clusters will be assessed after conducting training sessions and will be ratified accordingly if needed.
- Standard procedures will be applied in conducting anthropometry.
- Filled questionnaires will be checked for completeness and consistency of information by the supervisors on daily basis.
- Daily supervision in every step of data collection
- To enhance blinding, precise objectives of the study and village allocation to trial will not be disclosed to data collectors, HDA leaders will not be responsible for data collection, and the data collection schedule will be randomized.

**Data management and statistical and analyses**

Data will be checked, coded and double entered using EPI info and exported to SPSS version 21 for statistical analysis. Baseline differences between the study groups were tested using the chi-square test for categorical variables and *t*-test for continuous variables. Linear mixed models and generalized estimating equations adjusted for covariates and clustering will be used to test the intervention effects for continuous and categorical variables, respectively. Survival analysis will be conducted to compare the time of initiation of complementary foods in the control and intervention groups. All analyses will be conducted according to the intention to treat principle and values will be considered as statistically significant at P-value <0.05.

**Operational definitions**

- Health status: infant morbidity within two weeks before the study visit [25].
- Time of initiation of complementary food: the age at which mothers first initiated any solid, semi-solid, or soft foods to the index child in addition to breast milk. If the mother initiated complementary food for the child before 6 months, it was categorized as “early initiation of complementary food”; if she had initiated at 6 months, it was categorized as “timely initiation of complementary food” and if she had initiated after 6 months, it was categorized as “late initiation of complementary food” [42].
- Minimum dietary diversity: the proportion of children 6-23 months of age who receive foods from four or more food groups during the previous day [42].
- Minimum meal frequency: the proportion of children 6-23 months of age who receive solid, semi-solid or soft foods the minimum number of times or more (minimum is defined as two times for breastfed infants 6-8 months; three times for breastfed children 9-23 months) in the previous day [42].
- Minimum acceptable diet: the proportion of children 6-23 months of age who had at least the minimum dietary diversity and the minimum meal frequency during the previous day [42].
- Growth: an increase in height or weight of infants [21].
- **Stunting:** below minus two standard deviations from the median height-for-age of reference population [21].
- **Underweight:** below minus two standard deviations from the median weight-for-age of reference population [21].
- **Wasting:** below minus two standard deviations from the median weight-for-height of reference population [21].

**Ethical considerations and permissions**

All procedures involving the research will be approved by Jimma University College of Health sciences institutional and review board (IRB). Permission to undertake the study will be obtained from the regional, zonal and district administration and health offices of the study area. After the identification of eligible mothers, the nature and purpose of the study will be explained along with their right to refuse. Written informed consent will be obtained from all study participants**.** The right of the participant to withdraw from the study at any time will be respected. The data will not be accessed by a third person, except investigators, and will be kept confidential.

**References**

1. Black RE, Victora CG, Walker SP, Bhutta ZA, Christian P, de Onis M, et al. Maternal and child undernutrition and overweight in low-income and middle-income countries. Lancet 2013; 382(9890):427-51.
2. Rice AL, Sacco L, Hyder A, and Black RE: Malnutrition as an underlying cause of childhood deaths associated with infectious diseases in developing countries. WHO 2000; 78(10):1207-21.
3. Victora CG, Adair L, Fall C, Hallal PC, Martorell R, Richter L, et al. Maternal and child undernutrition: consequences for adult health and human capital. Lancet 2008, 371(9609):340–57.
4. Stevens GA, Finucane MM, Paciorek CJ, Flaxman SR, White RA, Donner AJ, Ezzati M: Trends in mild, moderate, and severe stunting and underweight, and progress towards MDG 1 in 141 developing countries: a systematic analysis of population-representative data. Lancet 2012, 380:824-834.
5. Black RE, Allen LH, Bhutta ZA, Caulfield LE, de Onis M, Ezzati M, et al: Maternal and child undernutrition: global and regional exposures and health consequences. Lancet 2008, 371(9608):243-260.
6. Central statistical agency of Ethiopia: Ethiopian mini-demographic and health survey 2014, Addis Ababa, Ethiopia.
7. Shrimpton R, Victora CG, de Onis M, Lima RC, Blossner M, and Clugston G: Worldwide timing of growth faltering: implications for nutritional interventions. Pediatrics 2001, 107(5):75.
8. Dewey KG, and Adu-Afarwuah S: Systematic review of the efficacy and effectiveness of complementary feeding interventions in developing countries. Maternal and Child Nutrition 2008, 4:24-85.
9. WHO. Infant and young child feeding 2009, Geneva, Switzerland.
10. Victora CG, Adair L, Fall C, Hallal PC, Martorell R, Richter L, et al. Maternal and child undernutrition: consequences for adult health and human capital. Lancet 2008;371(9609):340–57.
11. WHO/UNICEF/USAID/FANTA/IFPRI: Indicators for Assessing Infant and Young Child Feeding Practices: Part 1: Definitions 2008, Geneva, Switzerland.
12. WHO/UNICEF/USAID/FANTA/IFPRI. Indicators for Assessing Infant and Young Child Feeding Practices: Part 2: Measurement 2010, Geneva, Switzerland.
13. Kramer M.S; and Kakuma R: The Optimal Duration of Exclusive Breastfeeding: A Systematic Review 2001.
14. Victora CG, de Onis M, Lima RC, Blössner M, and Clugston G: Worldwide timing of growth faltering: implications for nutritional interventions. Pediatrics 2001, 107(5):75.
15. Kim SS, Ali D, Kennedy A, Tesfaye R, Tadesse AW, Abrha TH. Assessing implementation fidelity of a community- based infant and young child feeding intervention in Ethiopia identifies delivery challenges that limit reach to communities : a mixed-method process evaluation study. 2015;1–14.
16. USAID. Ethiopia-TIPS-report. 2011;(December). Available from: http://www.manoffgroup.com/6IYCN-Ethiopia-TIPS-report-120111.pdf.pdf
17. Ethiopia’s Community Health Workers 2010; 20 :2–4.
18. Negash C, Belachew T, Henry CJ, Kebebu A, Abegaz K, Whiting SJ. Nutrition education and introduction of broad bean–based complementary food improve knowledge and dietary practices of caregivers, and the nutritional status of their young children in Hula, Ethiopia. 2014;35(4):480–6.
19. Kang Y, Suh YK, Debele L, Juon H, Christian P. Effects of a community-based nutrition promotion programme on child feeding and hygiene practices among caregivers in rural Eastern Ethiopia. 2016;20(8):1461–72.
20. WHO: Report of Informal Meeting to Review and Develop Indicators for Complementary Feeding. Food and Nutrition Program Regional Office for the Americas Washington, D.C: World Health Organization; 2002.
21. PAHO/WHO: Guiding Principles for Complementary Feeding of the Breastfed Child. Pan American Health Organization/World Health Organization 2002, Washington DC. 16
22. Ministry of Human Resource Development, Department of Women and Child Development, Food and Nutrition Board. National Guidelines Infant and Young Child Feeding Practices 2004, Government of India, New Delhi.
23. Kibebew A: Infant and Young Child Feeding Practices among mothers Living Harar Town. Harar Bulletin of Health Sciences 2012,4: 66-78.
24. Jones, G: How Many Child Deaths Can We Prevent This Year? The Lancet 2003 362, 65-71.
25. WHO/UNICEF: Infant and Young Child Feeding Counseling: An Integrated Course. Participant’s Manual 2006.
26. Central statistical agency of Ethiopia: Ethiopia Demographic & Health Survey 2011, Addis Ababa, Ethiopia.
27. Guldan GS, Fan HC, and Ma X: Culturally appropriate nutrition education improves infant feeding, and growth in rural Sichuan, China. Journal of Nutrition 2000, 130:1204-11.
28. World Health Organization. Global strategy for infant and young child feeding, annex 2. Geneva: World Health Organization, 2002.
29. WHO: Implementing the global strategy for infant and young child feeding 2003. Available from http://whqlibdoc.who.int/publications/2003/924159120X.pdf.
30. Family Health Department: National Strategy for Infant & Young Child Feeding Federal Ministry of Health 2004, Addis Ababa, Ethiopia.
31. Tessema M, Kebede A, Hailu T, Kuche D, Samuel A, Assefa T. Implementation of the community-based nutrition program in Ethiopia after integrated refresher training technical report phase two, Ethiopian Health and Nutrition Research Institute, Food, and Nutrition Research Directorate 2013, Addis Ababa, Ethiopia.
32. Amhara Regional State. Budget Brief Amhara Regional State 2007/08 – 2015/16. Unicef. 2016;
33. Action IFOR. Federal Democratic Republic of Ethiopia. Pplicy and Practice. 2015;5(1).
34. Maes KC, Tesfaye YA. A Women’s Development Army: Narratives of Community Health Worker Investment and Empowerment in Rural Ethiopia 2015;(September).
35. MOH. The Federal Democratic Republic of Ethiopia. Health Sector Development Program IV 2010/11-2014/15, (October 2010).
36. Summary E, Bissau G-. UNICEF Annual Report 2013 – Ethiopia UNICEF Annual Report 2013 – Ethiopia. 2018;(September 2012):1–62.
37. Bilal NK, Herbst CH, Zhao F, Soucat A. Health Extension Workers in Ethiopia: Improved Access and Coverage for the Rural Poor. 2005;433–44.
38. Teklehaimanot A. Human resource development for a community-based health extension program : a case study from Ethiopia. Hum Resour Health [Internet]. 2013;11(1):1. Available from: Human Resources for Health
39. Campbell MK, Piaggio G, Elbourne DR, Altman DG. Consort 2010 statement: Extension to cluster-randomized trials. BMJ. 2012;345(7881):1–21.
40. Dewey KG, Adu-Afarwuah S. Systematic review of the efficacy and effectiveness of complementary feeding interventions in developing countries. Matern Child Nutr. 2008;4(SUPPL.1):24–85.
41. Piwoz E, Baker J, Frongillo EA. Documenting large-scale programs to improve infant and young child feeding is key to facilitating progress in child nutrition. Food Nutr Bull. 2013;34(3):143–5.
42. WHO. Indicators for assessing infant and young child feeding practices. World Heal Organ. 2007(November):1–19. Indicators for assessing infant and young child feeding practices. Available from <http://scholar.google.com/scholar>.
